# Topical neurokinin-1 receptor antagonism ameliorates ocular pain and prevents corneal nerve degeneration in an animal model of dry eye disease

**DOI:** 10.1097/PR9.0000000000001232

**Published:** 2025-01-17

**Authors:** Amirreza Naderi, Yukako Taketani, Shudan Wang, Francesca Kahale, Ann Yung, Pier Luigi Surico, Yihe Chen, Reza Dana

**Affiliations:** Department of Ophthalmology, Harvard Medical School, Schepens Eye Research Institute of Massachusetts Eye and Ear, Boston, MA, USA

**Keywords:** Dry eye disease, Neurokinin-1 receptor, Substance P, Eye-wiping test, Palpebral ratio, Trigeminal ganglion

## Abstract

**Introduction:**

Ocular pain is a common complaint to eye care providers, associated with a variety of ocular conditions, among which dry eye disease (DED) is affecting millions of people worldwide. Despite being highly prevalent, ocular pain is not managed adequately in the clinic.

**Objectives:**

The aim of this study was to investigate the analgesic potential of neurokinin-1 receptor (NK1R) antagonism in DED.

**Methods:**

Dry eye disease was induced in mice, and an NK1R antagonist L-733,060 was topically administered twice daily throughout the study for 14 days. Hyperalgesia and allodynia were assessed using the eye-wiping test and palpebral ratio measurements. Corneas were collected for measuring substance P (SP) levels by enzyme-linked immunosorbent assay (ELISA) and imaging nerves by immunostaining. Trigeminal ganglions (TG) were collected to determine SP levels by ELISA and transient receptor potential cation channel subfamily V member 1 (TRPV1), transient receptor potential cation channel subfamily M (melastatin) member 8, c-Fos, and activating transcription factor 3 (ATF3) mRNA levels by real-time polymerase chain reaction.

**Results:**

Treating DED mice with L-733,060 resulted in a significant reduction in eye wipe behavior, a significant increase in palpebral ratio, and significant decreases in SP levels in both the cornea and TG compared with the vehicle-treated group. In addition, NK1R antagonist treatment significantly suppressed the upregulation of TRPV1, ATF3, and c-Fos and prevented corneal nerve loss.

**Conclusion:**

Neurokinin-1 receptor antagonism effectively reduced ocular nociception, decreased neuronal activation, and preserved corneal nerves in mice with DED. These findings suggest that blockade of SP signaling pathway is a promising therapeutic strategy for managing DED pain.

## Abstract 2

Topical neurokinin-1 receptor antagonism suppresses dry eye–induced ocular hypersensitivity, corneal nerve loss, and trigeminal ganglion neuronal activation.

## 1. Introduction

Dry eye disease (DED) is one of the most common ocular conditions.^23^ It affects millions of people worldwide with an enormous economic burden, roughly equal to 4 billion dollars annually in the United States.^33,73,87^ Dryness and general ocular discomfort and pain are among the chief complaints of patients with DED.^1,68^ Consequently, patients' quality of life is substantially affected, limiting vision-related daily activities and productivity.^75^ According to one of the latest definitions, DED is a multifactorial disease characterized by the loss of ocular surface homeostasis and a variety of ocular symptoms. Tear film instability and hyperosmolarity, ocular surface inflammation and damage, and neurosensory abnormalities together play a crucial role in DED pathogenesis.^25^

While DED is generally classified into 2 categories, the aqueous-deficient form (eg, Sjogren syndrome) and the evaporative form (eg, meibomian gland dysfunction), the 2 entities are often overlapping in a setting of perpetuating inflammation,^3,15,26^ which promotes ocular surface nerves to release neuropeptides that in turn further amplify inflammation (neurogenic inflammation), leading to the vicious cycle of dry eye disease.^2^ Today, there is an increasing body of evidence highlighting the role of neuroimmune crosstalk in chronic painful conditions and autoimmune diseases where neuroimmune circuits amplify one another, leading to protracted inflammation and pain.^22^

The cornea is the most densely innervated tissue in the human body.^12^ Corneal nerves relay a variety of sensations including touch, temperature, and pain through their soma in the trigeminal ganglion (TG).^53^ These nerves also regulate tear production, blinking, and release of trophic factors.^8,61,70^ During neurogenic inflammation, corneal nerves produce neurotransmitters such as substance P (SP) to recruit and activate immune cells. In turn, the release of inflammatory mediators from immune cells sensitizes nociceptors and increases the release of SP, creating a self-perpetuating cycle.^9,39,78^ Substance P is an 11-amino acid long neuropeptide from the tachykinin family, encoded by *Tac1* gene. Neurokinin-1 receptor (NK1R) is the preferred receptor of SP and is expressed by a variety of cells including neurons, immune cells, keratocytes, and corneal epithelial cells and corneal nerves.^78,82^ Elevated levels of SP have been detected in tears of patients with DED ^12^ and cornea and TG of murine DED model compared with non-DED controls.^88^ The neuroimmune crosstalk through SP plays a pivotal role in DED pathogenesis through the induction and maintenance of ocular surface damage.^47,54^ The resulting imbalance of the ocular surface immune homeostasis leads to the activation of effector T cells causing disruptions of the tear film stability and corneal epithelial barrier.^20,21^ Substance P is a well-known mediator of nociception, contributing to inflammatory and neuropathic pain through various mechanisms.^18,19,24,45,46,51,59,60,62,63,65,66,82^ In addition, evidence of SP-driven stimulation of the corneal-trigeminal axis supports the centralization of pain response following ocular surface damage or chronic DED.^30,34,83^

Alteration in corneal innervation has been reported in DED patients with a correlation between reduced corneal nerve density and severity of DED.^25,71^ There is also evidence suggesting that decreased corneal nerve density is associated with DED-related neuropathic corneal pain.^37,58^

Previously, our group reported a significant reduction of corneal nerve sub-basal plexus following desiccating stress (DS) in a mouse model of DED.^52^ In the same animal model, modulation of SP signaling through the blockade of NK1R was shown to significantly reduce DS-induced inflammation by suppressing antigen presentation and resultant Th17 response, restoring the immunosuppressive capacity of dysfunctional Treg.^79,88^ The purpose of this study was to investigate the analgesic efficacy of NK1R antagonism in the DED model and its effect on corneal nerves.

## 2. Methods

### 2.1. Animals

C57BL/6 healthy female mice (6–8-week-old; Charles River Laboratories, Wilmington, MA) were housed in a climate-controlled, pathogen-free environment and kept under cyclic light conditions. All experiments were approved by the Institutional Animal Care and Use Committee at the Schepens Eye Research Institute and were conducted in accordance with the Association for Research in Vision & Ophthalmology Statement for the Use of Animals in Ophthalmic and Vision Research.

### 2.2. Dry eye disease induction

Dry eye disease was induced according to our previous studies.^88^ In brief, the animals were housed in a controlled environment chamber with low humidity (relative humidity < 15%), constant airflow of 15 L/min, and temperature of 21°C to 23°C for 14 days.

### 2.3. Topical treatments

Mice were assigned randomly to 1 of the 3 groups. The NK1R antagonist L-733,060 (R&D Systems, Minneapolis, MN) at 1 μg/μL or phosphate-buffered saline (PBS) as vehicle was administered 3 µL topically twice daily from day 0 to day 14. The treatment commenced nearly 12 hours after DED induction. Age-matched and sex-matched animals housed in room air conditions served as non-DED controls. The behavioral tests were done approximately 6 hours after instillation of the first drop on the day of assessment to minimize any potential confounding effect of ocular surface wetting on behavioral assays. All experiments used 5 animals per group unless stated otherwise.

### 2.4. Eye-wiping test

To evaluate hyperalgesia, eye-wiping test (EWT) was performed according to previous reports.^32^ In brief, mice (n = 10 animals/group) were habituated in an empty cage for 10 minutes. Eye wipes were counted for 30 seconds following instillation of hypertonic saline (2M NaCl) which served as a noxious stimulus.

### 2.5. Palpebral ratio

Palpebral ratio is the height (gap between the upper and lower eyelids)/width (distance separating the 2 canthi) ratio of palpebral fissure of eye. Decreased palpebral ratio (PR) for a long time has been considered a sign of ocular discomfort in rodents,^30,48,49,56,64^ and its value has been correlated to ocular allodynia when measured in response to innocuous stimuli,^49^ the lower the PR the more pain/discomfort. To evaluate allodynia, PR was quantified by analyzing 15 seconds videos of animals' eye (n = 10 animals/group) at 120 fps following instillation of normal saline (0.9% NaCl), which served as an innocuous stimulus, using DeepLabCut to measure the ratio of the height (distance of superior and inferior edges) and width (distance of nasal and temporal corners) of the palpebral fissure.

### 2.6. Dissection and tissue collection

Whole eyeballs were enucleated, and the corneas were isolated using microsurgery scissors. The TGs were isolated according to previously published methods without transcardial perfusion.^44^ In brief, the calvaria was first removed and the trigeminal ganglion was exposed and dissected ensuring the totality of the ophthalmic branch was taken. After collection, the tissues were transferred to appropriate buffers detailed below for subsequent analysis.

### 2.7. Enzyme-linked immunosorbent assay

Substance P levels of cornea and trigeminal ganglion (n = 5 animals/group) were quantified using Substance P Parameter Assay Kit (Catalog No. KGE007, R&D systems) according to the manufacturer's instruction. On day 14, both tissues were collected on ice directly into RIPA buffer containing (1:200) protease inhibitor cocktail (Catalog No. P8340, Sigma-Aldrich, St. Louis, MO) and homogenized the same day after harvest and stored in −80°C before enzyme-linked immunosorbent assay (ELISA).

### 2.8. Real-time polymerase chain reaction

The mRNA levels of c-Fos (Mm00487425_m1), activating transcription factor 3 (ATF3) (Mm00476032_m1), transient receptor potential cation channel subfamily V member 1 (TRPV1) (Mm01246300_m1), transient receptor potential cation channel subfamily M (melastatin) member 8 (TRPM8) (Mm01299593_m1), and glyceraldehyde 3-phosphate dehydrogenase (GAPDH) (Mm99999915_g1) were evaluated in trigeminal ganglion (n = 5 animals/group) using TaqMan Universal Polymerase Chain Reaction (PCR) Master Mix. In brief, the tissues were harvested on day 14 and stored in TRIzol reagent (Invitrogen, Carlsbad, CA) at −80°C until RNA was extracted using RNeasy micro kit (Qiagen, Valencia, CA) and reverse transcribed using SuperScript III kit (Invitrogen). Real-time polymerase chain reaction (RT-PCR) was performed for each gene in duplicates using Applied Biosystems StepOnePlus Real-time PCR system according to the manufacturer's protocol. In brief, the reactions were performed in a total reaction volume of 20 μL with 40 cycles of 60 seconds at 60°C, followed by 15 seconds at 95°C. The data were analyzed by the ∆C_t_ method after normalizing to GAPDH.

### 2.9. Immunohistochemistry

Eyeballs (n = 5 animals/group) were collected on day 14 and were immediately fixed in cold methanol at 4°C for 20 minutes. After 3x wash with PBS, corneas were dissected and placed in permeabilization buffer (0.5% Triton-X100 in PBS) for 1 hour at room temperature. After 3x wash with PBS, the corneas were blocked with a 0.3% PBS solution of Triton X-100 and 5% bovine serum albumin (BSA) in PBS for 2 hours at room temperature. After blocking, the tissues were stained with rabbit antimouse Alexa Fluor 488-conjugated β III Tubulin (Millipore Sigma, Burlington, MA) diluted in the blocking buffer (1:200) for 36 hours at 4°C. The corneas were then washed 5x in PBS before getting cut and flat mounted on a slide with the epithelium side up using VectaShield. Corneal whole mounts were imaged by a masked experimenter using confocal microscopy (Leica SP8 Laser Confocal Microscope). Corneal nerve fiber length (CNFL), the total length of all nerve fibers (µm) in the central corneal region (mm^2^, 200x microscope fields) were traced and quantified (µm/mm^2^) by the automatic filament detection feature of IMARIS (Oxford Instruments, Abingdon, United Kingdom). Reported values are averages of 3 images per cornea.

### 2.10. Statistics and software

All behavioral data and nerve density were from single eyes while all protein and mRNA data were from pooled paired cornea or trigeminal ganglions of a single animal. All data points represent biological replicates and are reported as mean ± standard error of mean. Palpebral ratio ratios were quantified by DeepLabCut.^55^ DeepLabCut was trained on 200 human-labeled images randomly taken from the videos. Each image was labeled with 4 points: superior, inferior, nasal, and temporal. The training error and test error were 4.2 and 5.8 px, respectively, and the nasotemporal distance was averaged at 60.488 px. All nerves were traced by the automatic filament detection algorithm of IMARIS. Enzyme-linked immunosorbent assay and RT-PCR data were analyzed in Microsoft Excel. All data were graphed and statistically compared using 1-way ANOVA with Tukey post hoc test in GraphPad PRISM 9, except the behavioral data of treatment vs vehicle, which were compared with multiple *t* tests in GraphPad PRISM 9.

## 3. Results

### 3.1. Dry eye disease mice experience hyperalgesia-like hypersensitivity that is suppressed by topical neurokinin-1 receptor antagonism

**Figure 1.**
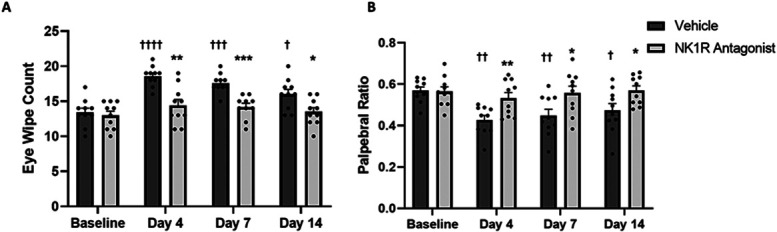
NK1R antagonism reduced hyperalgesia-like and allodynia-like ocular hypersensitivity in DED mice. (A) NK1R antagonism significantly reduced eye wipe behavior in response to noxious stimulus (indicating hyperalgesia) on days 4, 7, and 14 in DED mice. (B) Similarly, the treatment significantly decreased nociception in response to innocuous stimulus (indicating allodynia) on days 4 and 14 in DED mice. All data (n = 10/group) are reported as means ± standard error of mean. † denotes statistical significance compared with baseline and * denotes statistical significance between treatment and vehicle († and *P < 0.05, †† and **P < 0.01, ††† and ***P < 0.001, and ††††*P* < 0.0001). DED, dry eye disease; NK1R, neurokinin-1 receptor.

Dry eye disease mice demonstrated significantly increased eye wipe behavior to noxious stimulus (NaCl 2M) on all assessment days when compared with baseline, suggesting hyperalgesia (Fig. 1A). The eye wipe behavior peaked on day 4 (18.6 ± 0.45, *P* < 0.0001) and remained high on day 7 (17.6 ± 0.43, *P* < 0.001) and day 14 (16.0 ± 0.70, *P* < 0.05). Topical application of L-733,060 twice daily significantly reduced the eye wipe behavior of DED mice on all days when compared with the PBS-treated group (*P* < 0.0001) (Fig. 1A). The treatment group showed a gradual decrease in eye wipes from day 4 (14.4 ± 0.86 vs 18.6 ± 0.45, *P* < 0.01) and reached the baseline value on day 14 (13.5 ± 0.61 vs 16.0 ± 0.70, *P* < 0.05).

### 3.2. Dry eye disease mice experience allodynia-like hypersensitivity that is suppressed by topical neurokinin-1 receptor antagonism

Dry eye disease mice had significantly decreased PR in response to innocuous stimulus (NaCl 0.9%) on all days of examination when compared with the naive baseline, indicating allodynia (Fig. 1B). The animals treated with NK1R antagonist had an increased PR when compared with the PBS group on respective days with statistically significant difference on days 4 (0.53 ± 0.03 vs 0.43 ± 0.02, *P* < 0.01), 7 (0.56 ± 0.03 vs 0.45 ± 0.03, *P* < 0.05), and 14 (0.57 ± 0.02 vs 0.47 ± 0.03, *P* < 0.05).

### 3.3. Topical neurokinin-1 receptor antagonism reduces expression of substance P, nociceptive channels, and markers of neuronal activation after dry eye disease induction

**Figure 2.**
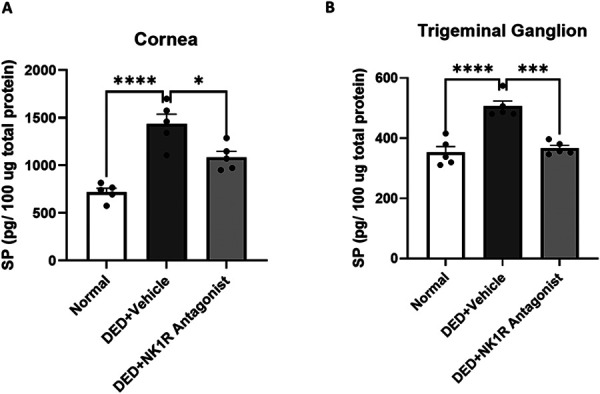
NK1R antagonism suppressed substance P levels in both the cornea and trigeminal ganglion (TG) in DED mice. (A) SP levels were significantly elevated on day 14 in the cornea of DED mice and treatment significantly reduced it. (B) Similarly, central expression of SP in the TG was significantly increased on day 14 and NK1R antagonism significantly decreased it. All data (n = 5/group) are reported as means ± standard error. * denotes statistical significance between groups (*P < 0.05, ***P < 0.001, ****P < 0.0001). DED, dry eye disease; NK1R, neurokinin-1 receptor; SP, substance P.

**Figure 3.**
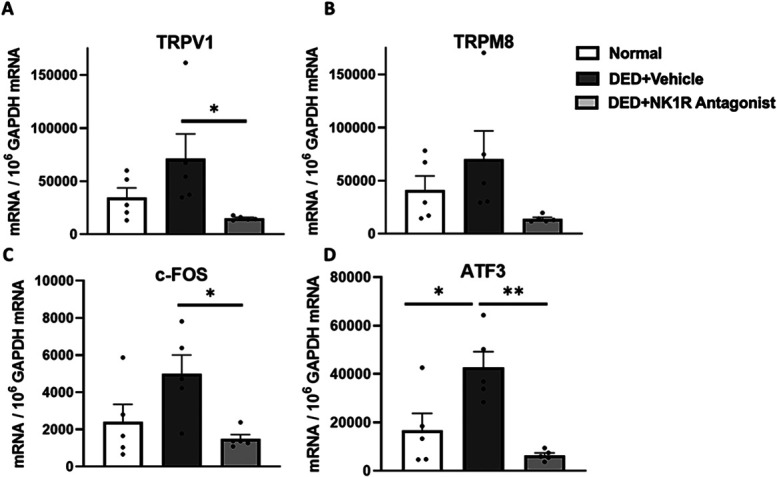
Topical NK1R antagonist suppressed transcription of nociceptive channels and markers of neuronal activation and injury in TG of DED mice. (A) NK1R antagonist significantly reduced expression of TRPV1. (B) Treatment also decreased TRPM8 expression, though this was not statistically significant. (C) Desiccating stress increased c-Fos, and NK1R blockade significantly lowered its transcription. (D) ATF3 transcription was significantly increased in DED, and NK1R antagonist significantly reduced its expression. All data (n = 5/group) are reported as means ± standard error. * denotes statistical significance between groups (*P < 0.05, **P < 0.01). ATF3, activating transcription factor 3; DED, dry eye disease; NK1R, neurokinin-1 receptor; TG, trigeminal ganglion.

Substance P levels of DED mice were significantly elevated on day 14 when compared with naive animals both in cornea (14362.71 ± 1017.2 vs 7185.95 ± 407.9 pg/mg, *P* < 0.0001) and TG (5057.41 ± 178.34 vs 3525.91 ± 196.75 pg/mg, *P* < 0.0001). Topical treatment with the NK1R antagonist significantly decreased SP levels in the cornea (10858.60 ± 614.51 vs 14362.71 ± 1017.22 pg/mg, *P* < 0.001) and TG (3663.62 ± 95.51 vs 5057.41 ± 178.34 pg/mg, *P* < 0.001), suggesting a negative feedback loop (Fig. 2). Furthermore, the TG of vehicle-treated DED mice expressed higher mRNA levels of TRPV1 (heat receptor, 2-fold increase) and TRPM8 (cold receptor, 1.7-fold increase) when compared with naive animals, and NK1R antagonist treatment reduced the expression levels of both channels by near 5 folds from vehicle-treated DED (*P* < 0.05 for TRPV1, and *P* = 0.08 for TRPM8) (Figs. 3A and B). In addition, PBS-treated DED showed upregulated transcriptions of ATF3 (*P* < 0.05), a marker of neuronal injury, and c-Fos, a neuronal activation marker, in the TG in comparison with naive animals. Blockade of SP signaling significantly reduced the transcriptive levels of ATF3 (6-folds decrease, *P* < 0.01) and c-Fos (3-folds decrease, *P* < 0.05) when compared with the vehicle group (Figs. 3C and D).

### 3.4. Topical neurokinin-1 receptor antagonism prevents corneal nerve loss

**Figure 4.**
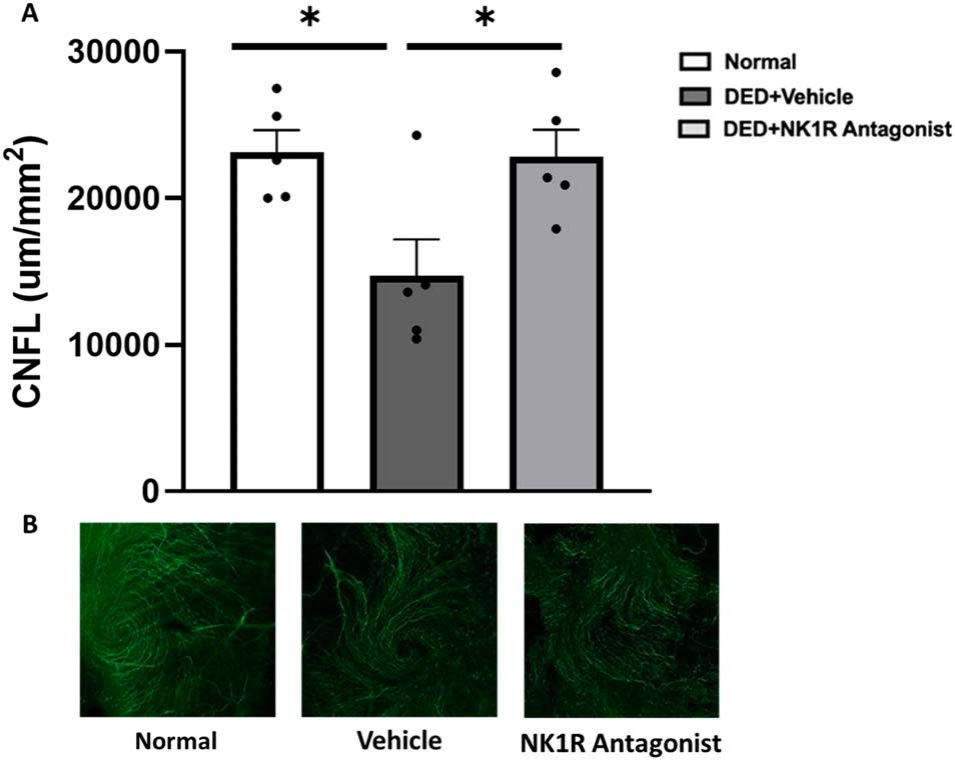
NK1R antagonism suppressed corneal nerve loss in DED mice. (A) Desiccating stress significantly decreased corneal nerve fiber length (CNFL) in central cornea. Topical treatment with an NK1R antagonist significantly reduced decrease of CNFL in the central cornea after 14 days. (B) Representative confocal images of central sub-basal nerves from individual groups. All data (n = 5/group) are reported as means ± standard error. * denotes statistical significance between groups (*P* < 0.05). DED, dry eye disease; NK1R, neurokinin-1 receptor.

Immunohistochemical analysis of corneal nerves revealed significantly lower CNFL in central corneas in comparison with naive animals (14676.75 ± 2509.25 vs 23160 ± 1490.84, *P* < 0.001). Neurokinin-1 receptor antagonist treatment prevented corneal nerve loss, indicated by the significantly higher nerve density in the central cornea (14676.75 ± 2509.25 vs 22820 ± 1863.71 µm/mm^2^, *P* < 0.001) (Fig. 4).

## 4. Discussion

Ocular discomfort/pain is by far the most burdensome symptom of dry eye disease, affecting patients' quality of life,^69^ and yet, there is no safe or effective treatment of DED pain; topical anesthetics, such as tetracaine, are highly effective for transient management of pain, but their administration is for limited periods due to significant side effects such as delayed reepithelization^31^ toxic keratopathy, corneal melting, and perforation.^84^ Oral medications such as nonsteroid anti-inflammatory drugs (NSAIDs) and opioids can also control ocular pain. However, oral NSAIDs come with significant side effects such as gastrointestinal disturbance, cardiovascular adverse effects, and renal toxicity.^11^ Opioids which are the most effective for pain come with serious side effects such as sedation, dizziness, nausea, vomiting, constipation, respiratory depression, tolerance, and, most importantly, physical dependence and addiction.^76^ Last, topical ophthalmic NSAIDs, such as diclofenac, are commonly used in the treatment of corneal inflammation and pain, though they are inherently less effective than topical anesthetics for pain. Frequently reported adverse reactions of topical NSAIDS include transient burning, stinging, corneal anesthesia, and ocular irritation on instillation.^43^ The continued use of topical NSAIDs may result in epithelial breakdown, corneal thinning, erosion, ulceration, or perforation.^35,36^

The analgesic efficacy of NK1R antagonists has been under investigation for years in both clinical and preclinical studies of various painful pathological conditions. Despite the efficacy of NK1R antagonists in preclinical studies, clinical trials have had mixed results. Although NK1R antagonists were not capable of suppressing nociceptive or neuropathic pain in several studies,^38^ they proved to be efficacious in a clinical trial to manage dental pain, which also is relayed through the trigeminal ganglion.^27^ The translation of drugs from bench to bedside is affected by many factors beyond interspecies differences. The systemic route of administration and bioavailability of the drug at target sites, the brain and spine, and different distribution of NK1R in different tissues could all contribute to the debate on NK1R analgesic efficacy.^38^ Most recently, there has been a case series report of effective treatment of 3 patients with neuropathic eye pain of different etiologies by NK1R antagonists.^77^ Neurokinin-1 receptor antagonists continue being rigorously investigated for a variety of indications including pain, inflammation, itching, and others (ClinicalTrials.gov). Among them, there are 3 ongoing studies assessing the analgesic effects in painful pathologies (NCT06317064, NCT02347878, NCT00775281) and 1 study planning to evaluate effects of NK1R antagonism on inflammation and pain in rare eye diseases (NCT06412718).

Insults to the ocular surface trigger axonal reflex inducing blinking and tear production on the ocular surface as a protective response. The peripheral axon terminals of corneal nerves propagate action potentials centrally to the TG to relay the sensation of pain from the insult; in addition, propagation of action potentials into other nerve terminal branches evokes the release of SP.^9,40^ Although from an evolutionary standpoint such as reflex and pain have a protective function, recurrent or chronic insults to the corneal nerves (as occurs in DED) can cause corneal nociceptors to undergo a variety of changes that lead to altered pain perception.^17,67^ Beyond pain, SP and NK1R signaling are involved in vasodilation and mediation of chemokine release that can mobilize numerous leukocyte/inflammatory cells.^72^ In turn, the release of inflammatory mediators can further sensitize nociceptors to actively release neuropeptides such as SP and amplify pain perception.^4–6^

We have previously shown that NK1R antagonism ameliorated corneal epitheliopathy in the same mouse DED model by restoring Treg function^79^ and suppressing antigen presentation and resultant Th17 response.^88^ This study highlights temporal changes in DED-associated hyperalgesia and allodynia and NK1R antagonism's analgesic efficacy in treating DED pain. Bignami et al.^10^ originally reported the analgesic efficacy of NK1R antagonists in a corneal alkali burn murine model and our group was the first to confirm this effect^80^ and demonstrate the anti-inflammatory properties of NK1R antagonism in DED.^88^ Later, independent groups reported similar findings, supporting our hypothesis. These proof-of-concept studies found a similar effect in eye wipe behavior, though reported at a single time point only, along with preservation of corneal nerve fibers when benzalkonium chloride–induced DED model and alkali burn injury model animals were treated with an NK1R antagonist.^13,14^ It is difficult to directly compare and comment on the difference in the eye wipe behavior and CNFL results of these studies with our findings due to multiple sources of variation. Beyond differences in the models, treatment agents, and timelines, there are significant differences in methodologies.

Measurement of animal behavior with predictive value particularly for studies of ocular pain is a challenging task. Decreased PR for a long time has been considered a sign of ocular discomfort in rodents,^30,48,56,64^ and its value has been correlated to allodynia^50^ (excessive pain perception from normally innocuous stimuli). However, analysis of slit-lamp images makes PR measurement partially subjective and laborious. We used here, for the very first time, artificial intelligence to quantify PR analyzing over 3600 frame/animal/time point. Interestingly, the ocular hypersensitivity observed in this study correlates temporally with previously published SP levels.^88^ Increased SP expression in tear samples from patients with DED^12^ and other models of DED^16^ further highlight SP/NK1R signaling as a key target to treat ocular pain in dry eye disease. Moreover, the decrease of SP expression in cornea and TG following treatment with NK1R antagonist was seen in intact murine cornea along reduced nociception, corroborating the negative feedback loop regulation of SP and its role in ocular pain.

Regarding the gene expression changes,^76^ it is plausible that certain mean values did not reach statistical significance since they were from the whole TG instead of the cornea-innervating neurons. Nevertheless, the findings of this study are in accord with the literature. There are reports of overexpression of TRPM8 in DED, which has been the principal theory for explaining the cold allodynia of patients with dry eye. In addition, TRPV1 upregulation has been proposed as one of the principal sensitization mechanisms in chronic pain conditions.^74,85^ In fact, enhanced TRPV1 expression at RNA and protein levels of different preclinical models of DED has been reported before.^7,28,29^ Moreover, TRPV1 has been shown to be required for the release of SP from TRPM8^+^ corneal nerves. According to this study, both TRPM8 and TRPV1 were increased, though not statistically significant, in the TG of DED mice. While it is difficult to compare the present findings to those of the past publications due to differences in model and methodology, the data underscore the reduction of TRPM8 and TRPV1 expression as a plausible mechanism through which NK1R antagonism suppresses ocular pain. The significant decrease in c-Fos expression at the transcription level following NK1R antagonism supports the behavioral findings since c-Fos is the preferred marker to assess the antinociceptive property of analgesic agents at a molecular level.^57,89^ Finally, DED animals had higher levels of ATF3 expression in their TG, indicating neuronal injury.^81^ In accordance with transcriptomic changes, corneal nerve fiber density significantly decreased in the DED animals. Assessment of corneal nerve fiber length (CNFL) was previously challenging due to its subjectivity and labor-intensive process, herein which was resolved by the employment of IMARIS.

Substance P is hypothesized to play a pivotal role in tear secretion and blinking reflex; however, the precise mechanism of NK1R signaling in release of tear from lacrimal gland is yet to be explored. From a clinical perspective, a decrease in sensory input from the eye surface is believed to increase the risk of DED in 2 ways. First, it reduces the reflex that triggers tear secretion, and second, it decreases the blink rate, leading to greater tear evaporation. Consequently, decreased tear secretion can lead to pathologic alterations in corneal nerves and a decline in corneal sensitivity, perpetuating the dry eye state. Furthermore, reduced corneal nerve density has been correlated with the severity of DED and pain.^25,71^ Surprisingly, NK1R antagonism had a neuroprotective effect and prevented corneal nerve loss despite intrinsic neuroregenerative properties of SP.^41,42,86^ A plausible explanation for this observation is suppression of the excessive SP-mediated inflammatory response that damages the central plexus. In the future, we hope to assess corneal-innervating neurons' activity for a more accurate measurement of pain response and mechanism through channel-specific stimulation. In summary, this study characterizes ocular pain and discomfort in a well-established model of DED and the effect of topical NK1R antagonism in suppressing DED-associated ocular pain along with the potential mechanisms.

## Disclosures

Massachusetts Eye and Ear owns intellectual property related to antinociception of targeting substance P in ocular surface diseases, and R.D. and Y.C. are co-inventors of this intellectual property.
